# Distribution of GABAergic Interneurons and Dopaminergic Cells in the Functional Territories of the Human Striatum

**DOI:** 10.1371/journal.pone.0030504

**Published:** 2012-01-17

**Authors:** Javier Bernácer, Lucía Prensa, José Manuel Giménez-Amaya

**Affiliations:** 1 Laboratorio de Neuromorfología Funcional, Clínica Universitaria de Navarra, Pamplona, Spain; 2 Departamento de Anatomía, Histología y Neurociencia, Facultad de Medicina, Universidad Autónoma de Madrid, Madrid, Spain; University of Chicago, United States of America

## Abstract

**Background:**

The afferent projections of the striatum (caudate nucleus and putamen) are segregated in three territories: associative, sensorimotor and limbic. Striatal interneurons are in part responsible for the integration of these different types of information. Among them, GABAergic interneurons are the most abundant, and can be sorted in three populations according to their content in the calcium binding proteins calretinin (CR), parvalbumin (PV) and calbindin (CB). Conversely, striatal dopaminergic cells (whose role as interneurons is still unclear) are scarce. This study aims to analyze the interneuron distribution in the striatal functional territories, as well as their organization regarding to the striosomal compartment.

**Methodology/Principal Findings:**

We used immunohistochemical methods to visualize CR, PV, CB and tyrosine hydroxylase (TH) positive striatal neurons. The interneuronal distribution was assessed by stereological methods applied to every striatal functional territory. Considering the four cell groups altogether, their density was higher in the associative (2120±91 cells/mm^3^) than in the sensorimotor (959±47 cells/mm^3^) or limbic (633±119 cells/mm^3^) territories. CB- and TH-immunoreactive(-ir) cells were distributed rather homogeneously in the three striatal territories. However, the density of CR and PV interneurons were more abundant in the associative and sensorimotor striatum, respectively. Regarding to their compartmental organization, CR-ir interneurons were frequently found in the border between compartments in the associative and sensorimotor territories, and CB-ir interneurons abounded at the striosome/matrix border in the sensorimotor domain.

**Conclusions/Significance:**

The present study demonstrates that the architecture of the human striatum in terms of its interneuron composition varies in its three functional territories. Furthermore, our data highlight the importance of CR-ir striatal interneurons in the integration of associative information, and the selective role of PV-ir interneurons in the motor territory. On the other hand, the low density of dopaminergic cells casts doubts about their role in the normal human striatum.

## Introduction

The basal ganglia are considered an important subcortical center for the integration and control of many high level cognitive [1], motor [2] and limbic [3] processes. It is well known that the striatum of primates (caudate nucleus, CN, and putamen, Put) receives associative, motor and limbic projections in different territories segregated throughout its whole extension. Thus, nearly the whole CN and precommissural Put are considered the associative part of the striatum. The dorsolateral rim of the CN and the postcommissural Put constitute the sensorimotor territory, and the only striatal region (excluding the nucleus accumbens) that contains limbic information is the posteroventral Put [4], [5], [6]. In addition, there are other striatal regions where these types of information overlap [3], [7].

In order to achieve a proper holistic response to certain stimuli, the neural information that reaches the various striatal territories must be integrated at two different levels: within each ‘channel’, and between them. Striatal interneurons are one of the mechanisms through which this integration takes place. In the last years, many studies have shown that this integrative role may be achieved at a single cell level (see, for example, [8], [9], [10], [11]). However, descriptive analyses about the actual presence of the different types of neurons in the human striatum are scarce [12], [13], [14], [15], [16] and, to our knowledge, only our group has included the three functional territories in the whole extension of the CN and Put in previous reports [17], [18].

The human striatum contains two different types of neurons respecting to the target of their axons: projection neurons, which represent about the 74% of the total neuronal population, and interneurons, which account for the remaining 26% [19], [20]. Whereas projection neurons are spiny and send their axons to the internal and external segments of the globus pallidum and to the substantia nigra *pars reticulata* [21], interneurons do not contain synaptic spines and exert a powerful control over projection neurons [22], [23]. Thus, a balanced activity of both neuronal populations is essential for the striatum to perform properly its cognitive, motor and limbic functions [3], [6], [7]. Six different groups of interneurons have been described in the human striatum to date: 1) nitrergic; 2) cholinergic; 3) calretinin-immunoreactive (-ir); 4) parvalbumin-ir; 5) calbindin (CB)-ir, and 6) dopaminergic interneurons [12], [16], [18], [24].

As a further step in the anatomical complexity of the striatum, the CN and Put are divided into two different chemical compartments: the matrix and the striosomes [25], [26], [27], [28]. Both interdigitating territories are not only neurochemically distinct, but also display a differential afferent and efferent connectivity [28], [29], [30], [31], [32]. Furthermore, it has been reported that the striosomal compartment is chemically heterogeneous in itself, showing a center or core and a periphery [27]. However, some striosomes can be homogeneous, especially in the posterior part of the striatum [33]. One of the best markers to distinguish the different striatal compartments is the neuropeptide enkephalin (ENK), which presents a stronger labeling in the striosomal periphery than either in the center of striosomes or in the matrix [18], [27], [34].

Given the mosaic-like functional organization of the striatum, one or several elements are required to control and integrate the incoming and outgoing neural information. Interneurons have been proved to be in charge of this role in several processes [35], [36]. Therefore, an exhaustive study of the anatomical arrangement of these cells in the human striatum would contribute to understand the functional organization of this key brain area.

The present study aims to seek for possible variations in the distribution of the different populations of GABAergic interneurons (CR-, PV- and CB-ir) and the tyrosine hydroxylase (TH-ir) cells with respect to the various functional territories and compartments of the human striatum. Following our previous results on the nitrergic and cholinergic interneurons, we hypothesize a higher neuronal density in the associative territory of the striatum. All these data may help understand the cellular and functional organization of the CN and Put, and evaluate the effects of neurological and psychiatric disorders in the human striatum.

## Materials and Methods

### Tissue preparation

The postmortem human brain material used in the present study was obtained from eight adult individuals without clinical or pathological evidence of neurological or psychiatric disorders (Table 1). This tissue was kindly provided by the Banco de Tejidos Neurológicos de Navarra (Clínica Universitaria and CIB), the Departamento de Anatomía Patológica (Clínica Universitaria, Universidad de Navarra, Pamplona) and Hospital Ramón y Cajal (Madrid). At the time of the decease, the relatives of the patients were asked for authorization to perform the medical autopsy. Then, many medical samples were anonymized and kept in the hospital for research purposes. The biological samples of the present study were provided by these Departments after the approval of our specific project by the corresponding Ethical Committees of the hospitals where the samples were taken (Clínica Universitaria and Universidad Autónoma de Madrid). All the cases used in this study were obtained between 2001 and 2004.

**Table 1:** Clinical data on the human cases used in this study.

Case	Sex	Age (Years)	Postmortem delay (h)	Weight* (g)	Cause of death
1	Male	35	4	1250	Cardiac arrest
2	Female	58	6	1345	Gastrointestinal hemorrhage
3	Male	66	17	1385	Gastric adenocarcinoma
4	Male	67	4.5	1280	Bilateral pneumonia
5	Male	74	7	1090	Gastric sarcoma
6	Male	63	12	1441	Prostate carcinoma
7	Female	20	2	1100	Cystic fibrosis
8	Male	76	12	1400	Gastrointestinal hemorrhage

*Weight of the whole unfixed brain.

The tissue preparation has been extensively reported elsewhere (for details on the protocol, see [17], [18], [37]). Briefly, the brains were cut into thin blocks that were fixed in 4% paraformaldehyde for 10 days. After that, they were immersed in 15% sucrose at 4°C for at least another 7 days before cutting. Those brains which were not sliced immediately were stored in a mixture of 15% sucrose and 0.1% sodium azide. Samples were cut along the coronal plane with a freezing microtome into 50 µm-thick coronal sections that were serially collected in a cryoprotective solution.

### Immunohistochemistry procedures

We selected adjacent sections throughout the whole striatum (see *Subdivision of the striatum* in *Data analysis*) and used an indirect immunohistochemical technique to visualize CR-, PV-, CB- and TH-ir interneurons, as well as ENK as a marker of the striosomal compartment. The procedure was divided in six steps: antigen retrieval, endogenous peroxidase inactivation, incubation of the primary antibody, incubation of the secondary antibody, avidin-biotin complex (ABC) and development. The first two steps were identical for all the markers but ENK. The antigen retrieval improved the signal strength of the final staining, and was performed by placing the sections in phosphate buffer 0.1 M pH 7.4 (PB) at 80°C during 30 minutes. After letting cool down at room temperature, the slices were treated in a solution containing 50% ethanol (1∶3) and 3% H_2_O_2_ (2∶3) for 30 minutes to inactivate endogenous peroxidase activity, and then rinsed thoroughly with 0.1 M PB saline (PBS).

After the preparation of the tissue, the primary antibodies were incubated as follows:

1. CR: a polyclonal anti-CR antibody obtained in rabbit (SWant, Bellinzona, Switzerland) at a 1∶2500 dilution was incubated overnight. The antiserum against CR is produced in rabbits by immunization with recombinant human CR containing a 6-his tag at the N-terminal. The antibody reacts specifically with CR in tissue originating from human, monkey, rat, mouse, guinea pig, chicken and fish. This antiserum does not cross-react with CB or other known calcium binding-proteins, as determined by immunoblots and by its distribution in the brain (information provided by the manufacturer).
2. PV: a monoclonal anti-PV antibody obtained in mouse (Sigma, St. Louis, MO) was chosen as the best marker to label these interneurons, and was used overnight at a 1∶2500 dilution. This antibody recognizes PV in a Ca^2+^ ion-dependent manner. It does not react with other members of the EF-hand family such as calmodulin, intestinal calcium-binding protein, S100A2 (S100L), S100A6 (calcyclin), the α chain of S-100 (i.e. in S-100a and S-100ao), or the β chain (i.e. in S-100a and S-100b) (information provided by the manufacturer). Among other species, it reacts against human brain tissue.
3. CB: a monoclonal anti-CB antibody produced in mouse (Sigma) at 1∶2000 dilution was incubated overnight. This antibody is specific for CB-d-28k in humans. It does not react with other members of the EF-hand family, such as CB-D-9K, CR, myosin light chain, PV, S-100a, S-100b, S-100A2 (S100L) and S-100A6 (calcyclin). A weaker reactivity was observed with chicken CB-D-28K (information provided by the manufacturer).
4. TH: a monoclonal anti-TH antibody produced in mouse (Incstar, Stillwater, MN) at 1∶500 dilution was incubated for two days. This antibody is believed to have wide species cross-reactivity, because it recognizes an epitope in the mid-portion of the TH molecule where extensive species homology exists. It does not cross react with dihydropteridine reductase, dopamine-B-hydroxylase, phenylethanolamine-N-methyltransferase, phenylalanine hydroxylase or tryptophan hydroxylase using Western blot methods (information provided by the manufacturer).
5. ENK: a monoclonal anti-ENK antibody obtained in mouse (Medicorp, Montreal, Canada) was used at a 1∶50 dilution, for two days. This monoclonal antibody is secreted by a hybridoma formed by the fusion of a NSO/1 mouse myeloma cell with a spleen cell from a BALB/C mouse immunized against Leu^5^-ENK conjugated to bovine serum albumin. This antibody does not distinguish between Met^5^-ENK and Leu^5^-ENK in immunohistochemistry. It displays about 40% cross-reactivity with C-Terminal extended Met-ENK hexapeptides and 7% cross-reactivity with the extended heptapeptide (-Arg-Phe-OH), but does not recognize other endogenous peptides. In immunohistochemistry, the antibody recognizes all well established ENK-ir sites but does not bind to areas known to contain β-endorphin or dynorphin (manufacturer's technical information).

All the solutions of the primary antibodies also included 0.1% Triton X-100 and 2% of the appropriate normal serum (goat serum for anti-CR, and horse serum for the remaining antibodies).

After the incubation of the primary antibodies, the sections were rinsed in PBS and placed in the solution containing the biotinylated secondary antibodies for 90 min at room temperature. We used an anti-rabbit IgG made in goat (Vector Labs, Burlingame, CA) for the labeling of CR, and an anti-mouse IgG made in horse (Vector Labs) for the remaining. They were diluted in PBS with Triton X-100 and normal serum as stated above. Then, and after several rinses in PBS, all the sections were immersed for 90 minutes at room temperature in a 1∶125 avidin-biotin complex solution (ABC, Vector Labs) according to the method of Hsu et al. [38]. The sections were developed by placing them in a medium containing 0.05% 3,3′-diaminobenzidine tetrahydrochloride (DAB, Sigma) and 0.003% H_2_O_2_ (30%) in 0.05 M Tris buffer pH 7.6 at room temperature. The reaction was stopped by rinses in Tris buffer. Subsequently, the sections were rinsed thoroughly in PBS, mounted onto gelatine-coated slides and air-dried overnight. They were then dehydrated through passages in ascending grades of alcohol, cleared in xylene and covered with DPX mounting medium. Control sections were incubated omitting either the primary or the secondary antibodies.

To analyze the distribution of the interneurons regarding to the ENK-rich striosomes, double-labeling with CR and ENK, whose primary antibodies were raised in different species, were performed. In the case of the PV, CB, and TH interneurons, visualized with antibodies raised in mouse as it was the ENK antibody, we used adjacent sections in such a way that one was treated to reveal one of these populations of interneurons and the other was immunostained for ENK.

The double immunohistochemical labeling for CR and ENK was as follows. Briefly, we incubated both primary antibodies in the same solution for two days, using bovine serum albumin instead of horse or goat normal serum. After that, we continued the CR staining as mentioned above, including the development. Then, the sections were extensively rinsed in PBS and placed in a solution containing the specific secondary antibody for anti-ENK. After the incubation of the ABC complex, we developed the sections in a nickel-DAB solution (0.024% DAB, 0.295% nickel ammonium sulphate and 0.003% H_2_O_2_ 30%) until the striosomes could be seen and the labeling did not mask the CR-ir interneurons.

### Data analysis

#### Subdivisions of the striatum

The CN and the Put were respectively subdivided into five and three anteroposterior territories, as described in previous reports [18]: precommissural and postcommissural head, body, gyrus and tail of CN; precommissural, postcommissural and posteroventral Put. The nucleus accumbens, which was delimited from the dorsal striatum as described by Selden and colleagues [39], was not included in this study because it shows numerous hodological and compartmental differences with the dorsal striatal tissue and, therefore, other chemical markers are required to visualize its core and shell domains.

In addition, the head of the CN and the Put were divided into four quadrants: dorsomedial, ventromedial, dorsolateral and ventrolateral. Then, each striatal region was included in one of the three functional domains of the striatum as follows: 1) associative: dorsomedial sector of the CN head, CN body and CN gyrus; 2) sensorimotor: postcommissural Put, excluding its posteroventral aspect; and 3) limbic: posteroventral Put [5]. Please note that this functional division only includes those areas that receive a single type of information, in order to minimize the effect of overlapping projections with different neuronal densities.

The following three atlases of the human brain have been used: Schaltenbrand and Wahren [40], Mai et al. [41] and Nowinski et al. [42]. The sections were examined using a Nikon SMZ1500 stereomicroscope (Nikon, Melville, NY) and a Nikon Eclipse 80i microscope (Nikon) equipped with a camera lucida and computerized image analysis system with a DXM1200 digital camera (Nikon).

#### Volume of perikarya

A total of 1,557 CR-, 459 PV-, 535 CB- and 106 TH-ir cells were randomly selected with the optical dissector (see below) throughout the striatum of the eight cases included in this study, and the volume of their perikarya was measured by means of the nucleator. This stereological technique consists in placing two randomly-oriented planes over the perikarya of the cells and then allowing the software to calculate the volume of the cell bodies by establishing the sites at which these planes intersect the boundaries of the perikarya.

#### Neuronal distribution and cell count

The cell density was determined along the complete anteroposterior length of both the CN and Put following a stereological protocol described elsewhere [17], [18]. The sample fraction we chose produced about 20 coronal sections per brain and for each neuronal group.

Cell density was analyzed using the optical dissector, an unbiased stereological method [43], [44], [45], as described previously by Martin and colleagues [46] and our own group [17]. The area of the striatum to be analyzed was selected at 4x and the neurons were counted at 20x magnification, using an Olympus microscope (Olympus Optical Co. Europe GmbH, Hamburg, Germany). This microscope was connected to a JVC TK-C1380 video camera (JVC Spain, Barcelona, Spain) and supplied with a motorized stage connected to a Dell OptiPlex computer. We used the CAST package software (Visiopharm, Hørsholm, Denmark) to command the movement of the motorized stage along the XY axes and to provide an automatic selection of microscopic fields, which were then captured by the video camera and projected onto the monitor. This same software generates the dissector grid that was superimposed over the microscopic field projected into the monitor.

The volume of the dissector (Vdis) was calculated by multiplying the area of the dissector grid by the distance between the two focal planes, which were measured with a microcator (Heidenhain, Traureut, Germany) connected to the Z axis of the microscope stage. The meander sampling was done with the same fraction (3.25%) in every sector of each striatal territory analyzed. The use of this fraction let us analyze a maximum of 100 dissectors in the widest sectors (for example, in any of the four quadrants in which the precommissural and postcommissural head of the CN were subdivided) and one dissector in the smallest (some sections of the CN tail). The sum of the number of neurons contained in each dissector corresponded to the ΣQd- parameter and the neuronal density (Nv) was calculated with the formula Nv = ΣQd-/ΣVdis (cells/mm3).

#### Statistics

We estimated the mean±standard error of the mean (SEM), the normality and the homogeneity of variances with the values of neuronal densities and volume obtained in the various striatal territories. Since the variances of the samples were rather heterogeneous, the statistical differences in the distribution pattern and volume of these interneurons were calculated using the Kruskal-Wallis and ANOVA tests with post-hoc Tamhane procedure for multiple comparisons. Either Mann-Whitney U or t-test was used to compare two independent samples. Significant or highly significant differences were respectively considered as 0.05>P>0.01 or P<0.01.

#### Compartmental distribution

Double-labeled CR/ENK coronal sections, and adjacent slices immunostained either for PV, CB, or TH and ENK were used. In all the cases, the striosomes were either stained homogeneously for ENK or contained a poorly stained center surrounded by an ENK-rich periphery. The location of the neurons within the striosomes and surrounding matrix was drawn at 5x or 10x using a camera lucida. The drawings were scanned and rendered with Canvas (Deneba Systems Inc, Miami, FL) software.

We randomly selected a sample of striosomes and calculated the percentage of them that contained interneurons in the dark ENK-ir part, in the light ENK-ir center, in the border between the striosome and the matrix, or in the center/periphery boundary. Then, we compared those percentages between functional territories (associative, sensorimotor and associative+sensorimotor overlapping domain) and neuronal types (CR- and CB-ir interneurons) using Fisher's exact test.

## Results

### Volume of the cell bodies of each chemospecific type of interneurons in the associative, sensorimotor and limbic striatal territories

#### CR

**Figure 1:**
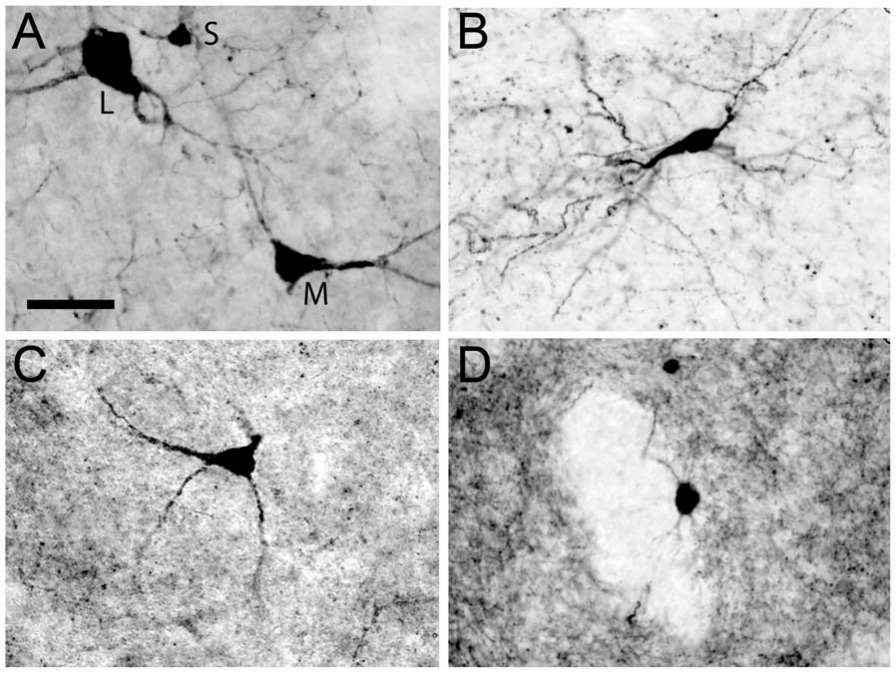
Photomicrographs showing the populations of interneurons included in the study. A, CR-ir (S, small, M, medium, L, large); B, PV-ir; C, CB-ir; and D, TH-ir cells. Calibration bar: 40 µm for all images.

**Figure 2:**
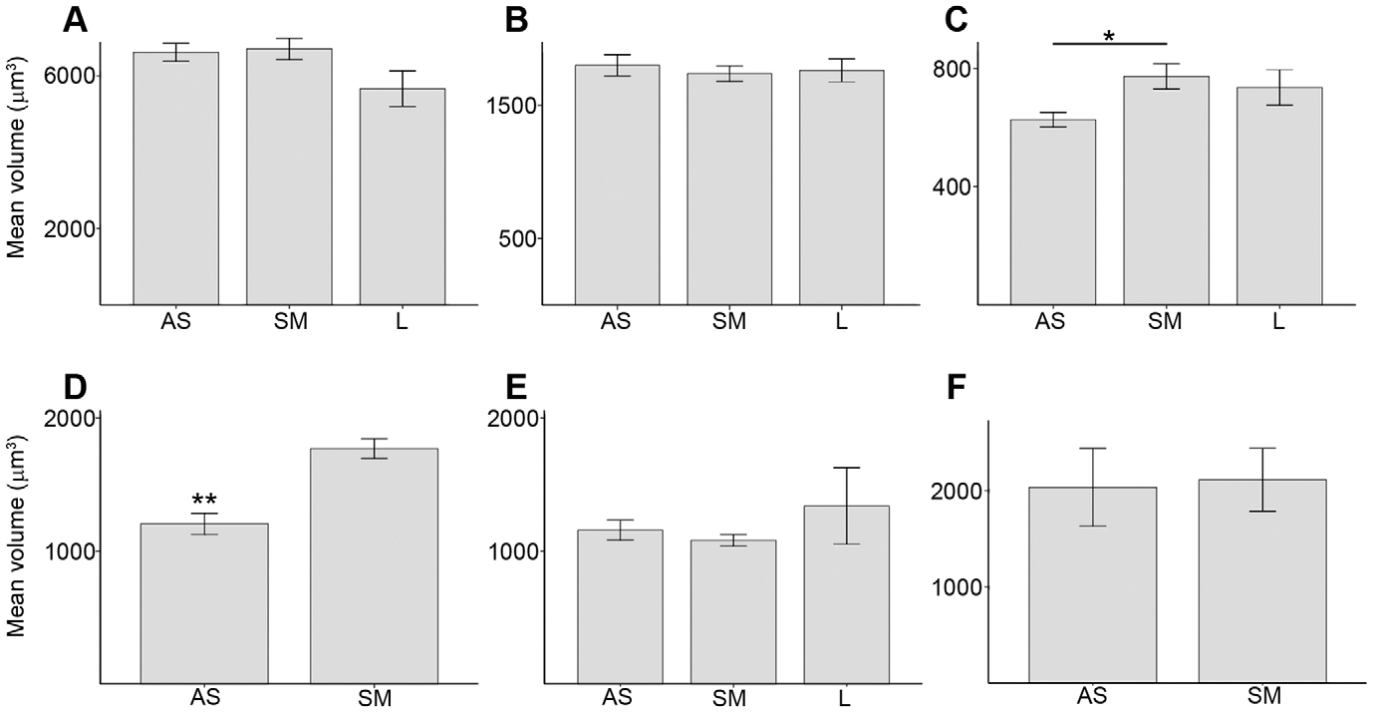
Mean volume of the soma. Bar graphs showing the mean volume of the soma for CR-ir (A, large; B, medium; C, small), PV-ir (D), CB-ir (E) interneurons and TH-ir cells in the human striatum. The only statistical differences were found for the small group of CR-ir cells (*, 0.05>P>0.01) and for PV-ir interneurons (**, P<0.01). Error bars indicate the SEM. AS, associative; L, limbic; SM, sensorimotor.

The immunohistochemical technique for the visualization of CR revealed a population of aspiny neurons clearly divided in three different groups, according to the size of their soma (large, medium and small; Figure 1A). The cell body of the large neurons was ovoid, fusiform or triangular, frequently with 3 or 4 primary dendrites emerging from it, and its volume averaged 6837±138 µm^3^. The shape of the medium group was similar, although its mean volume was clearly smaller (1767±37 µm^3^). The small CR-ir interneurons showed a rounded cell body whose volume averaged 662±13 µm^3^. The soma volume of large and medium neurons was rather homogeneous in the functional territories of the striatum (Figure 2A,B). However, the mean volume of small neurons was significantly lower in the associative territory, compared with the sensorimotor (ANOVA F(2,314) = 4.57, P = 0.011; Tamhane mean difference = −146.52, P = 0.01) (Figure 2C).

#### PV

The immunostaining for PV labeled a homogeneous subset of aspiny neurons with fusiform or oval soma from which 3 or 4 primary dendrites usually emerged (Figure 1B), and whose size averaged 1526±932 µm^3^. They were also significantly larger in the sensorimotor than in the associative territory (Mann-Whitney U = 3267.5, n1 = 70, n2 = 158, P<0.001) (Figure 2D).

#### CB

As it has been extensively reported, the CB immunostaining in the striatum revealed two different populations of neurons according to their labeling and general morphological features. The subset of cells with the darkest staining was considered interneurons, due to their morphology and aspiny dendrites. However, it is worth noting that the intensity of labeling followed a continuum from the palest to the darkest cells, and therefore some of them were difficult to include in one group or the other. In order to follow a reliable method, we have considered a cell as interneuron only when at least 2 aspiny primary dendrites emerging from the soma were visible and, in addition, one of them was subdivided in aspiny secondary dendrites (Figure 1C). CB-ir interneurons were similar to PV-ir cells in terms of morphology, although CB-ir ones were frequently triangular-shaped. They were smaller than PV-ir, their soma averaged 1183±25 µm^3^ and their volume values were rather homogeneous in the three different striatal territories (Figure 2E).

#### TH

Most of TH-ir cells had an oval soma, being fusiform a few of them (Figure 1D). Their primary dendrites were thick and intensely labeled, although they could be followed only for about 10 µm. Secondary dendrites were not usually seen in this subset of neurons. The mean size of their soma was 1896±1598 µm^3^, and it was homogeneous throughout the whole extension of the striatum (Figure 2F).

### Global distribution of the four types of interneurons in the associative, sensorimotor and limbic striatal territories

**Figure 3:**
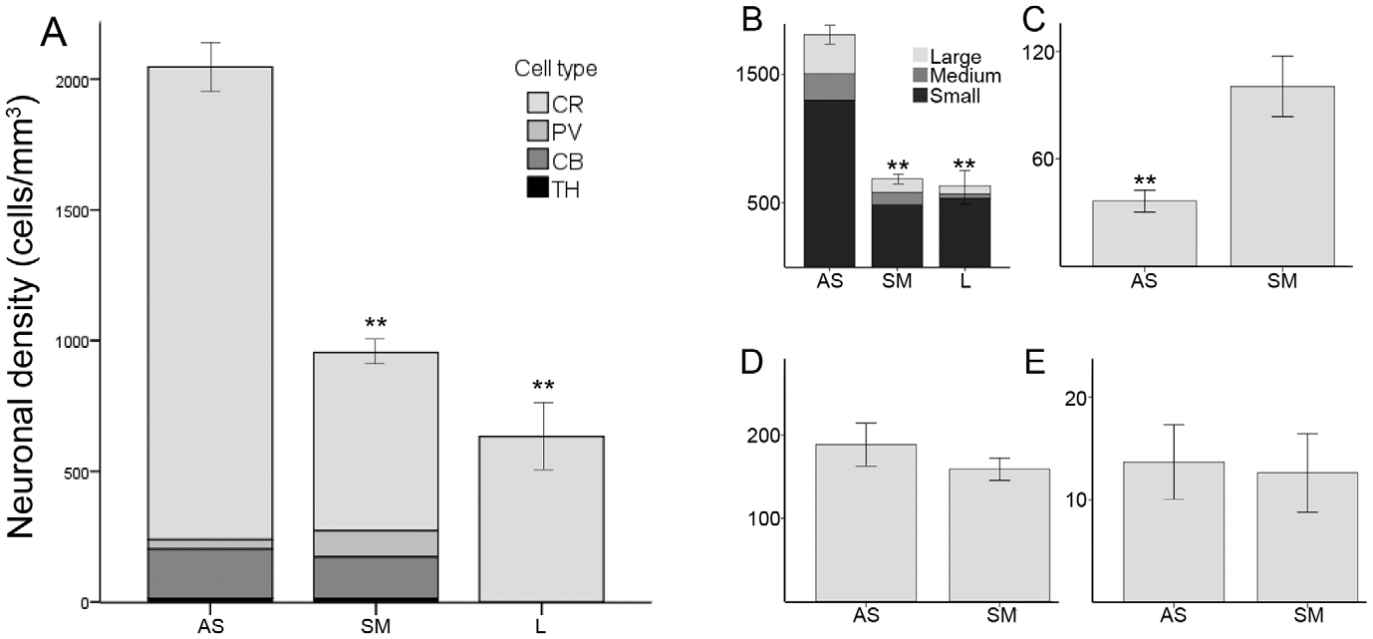
Neuronal density of the various groups of neurons in the functional territories of the striatum. A, stack bar graph showing the global interneuronal density. The colors of the stacks indicate different chemospecific subsets of neurons. B, stack bar showing the density (cells/mm^3^) of large, medium and small CR-ir interneurons. C–E, neuronal density (cells/mm^3^) of the PV-, CB- and TH-ir neurons, respectively. Error bars indicate the SEM. **, P<0.01. AS, associative; L, limbic; SM, sensorimotor.

As it has been described in the Materials and Methods section, as well as in previous reports [17], we only included striatal regions with a unique pattern of connections (either associative, sensorimotor or limbic). Therefore, the only limbic region was the posteroventral Put, whose cell density was possible to calculate only for the CR-ir interneurons. Considering the four groups of cells altogether, the density was higher in the associative (2120±91 cells/mm^3^) than in the sensorimotor (959±47 cells/mm^3^) or limbic (633±119 cells/mm^3^) territories. The statistical comparison between the three regions showed significant differences (ANOVA F(2,160) = 44.58, P<0.001), and the results were confirmed by the posthoc Tamhane test (P<0.001) for both associative vs sensorimotor (mean difference = 1160.66) and associative vs limbic (mean difference = 1490.52) (Figure 3A).

### Global distribution of the four types of interneurons in the associative/sensorimotor and associative/limbic overlapping striatal territories

**Figure 4:**
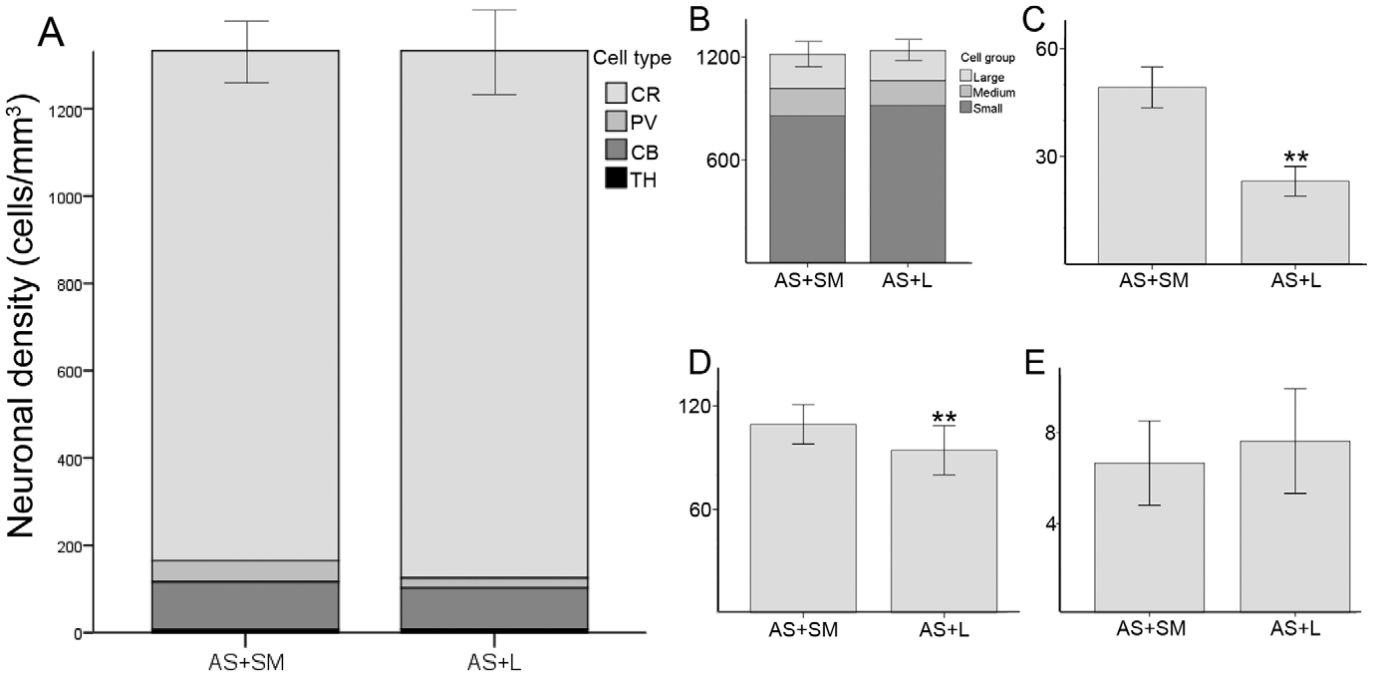
Mean neuronal density in the striatal territories with overlapped projections. A, stack bar graph showing the global interneuronal density (cells/mm^3^). The stacks indicate different groups of neurons. B, stack bar showing the density of CR-ir interneurons (cells/mm^3^) and their morphological subgroups. C–E, neuronal density of the PV-, CB- and TH-ir neurons, respectively (cells/mm^3^). Error bars indicate the SEM. **, P<0.01. AS+L, associative+limbic; AS+SM, associative+sensorimotor.

We have analyzed here striatal territories with overlapped connections, either associative plus sensorimotor (dorsolateral sector of precommissural and postcommissural head of CN, and dorsal half of precommissural Put), or associative plus limbic (ventral half of precommissural and postcommissural head of CN, tail of CN and ventral half of precommissural Put). The global distribution of the four chemospecific types of neurons included in this study was almost identical in both overlapped territories (1501±66 cells/mm^3^ for associative+sensorimotor, and 1502±99 cells/mm^3^ for associative+limbic) (Figure 4A).

### Distribution of each chemospecific interneuronal population in the functional striatal territories

#### CR

As it was expected, the distribution of CR-ir interneurons followed the same pattern as the global arrangement previously described. Hence, they specially occupied the associative territory (1808±79 cells/mm^3^), followed by the sensorimotor (684±37 cells/mm^3^) and limbic (633±119 cells/mm^3^) aspects (ANOVA F(2,188) = 44.77, P<0.001) (Figure 3B). Furthermore, the same trend was followed by the large-, medium- and small-sized CR interneurons (Figure 3B). The overlapped territories showed a very similar neuronal density when all the CR groups were taken together, being 1224±58 cells/mm^3^ in the associative/sensorimotor aspect and 1238±82 cells/mm^3^ in the associative/limbic. Within each morphological group, their densities were also comparable between both overlapped domains (Figure 4B).

#### PV

Interestingly, PV-ir interneurons showed an opposite pattern of distribution than the CR-ir ones. They were more abundant in the sensorimotor (101±20 cells/mm^3^) than in the associative (36±6 cells/mm^3^) striatum (Mann Whitney U = 778.5, n1 = 118, n2 = 31, P<0.001) (Figure 3C). Furthermore, considering the territories with overlapped projections, we also found significant differences (Mann Whitney U = 3892, n1 = 110, n2 = 109, P<0.001) between the associative+sensorimotor (49±6 cells/mm^3^) and the associative+limbic (23±4 cells/mm^3^) territories (Figure 4C).

#### CB

Unlike the two neuronal types already described, the distribution of CB-ir interneurons was rather homogeneous in the associative (189±26 cells/mm^3^) and sensorimotor (159±13 cells/mm^3^) striatum (Figure 3D). However, we did find statistical differences when comparing the associative+sensorimotor (109±12 cells/mm^3^) and the associative+limbic (94±14 cells/mm^3^) striatum (Mann Whitney U = 3555, n1 = 91, n2 = 105, P = 0.002).

#### TH

The TH-ir cells seemed to be evenly distributed in the associative (14±4 cells/mm^3^) and sensorimotor (13±4 cells/mm^3^) territories of the striatum (Figure 3E). Likewise, the analysis of the overlapped territories did not reveal any difference between the densities of the associative+sensorimotor (7±2 cells/mm^3^) and associative+limbic (8±2 cells/mm^3^) territories (Figure 4E).

### Distribution of CR- and CB-ir interneurons with relation to the ENK-rich striosomes

**Figure 5:**
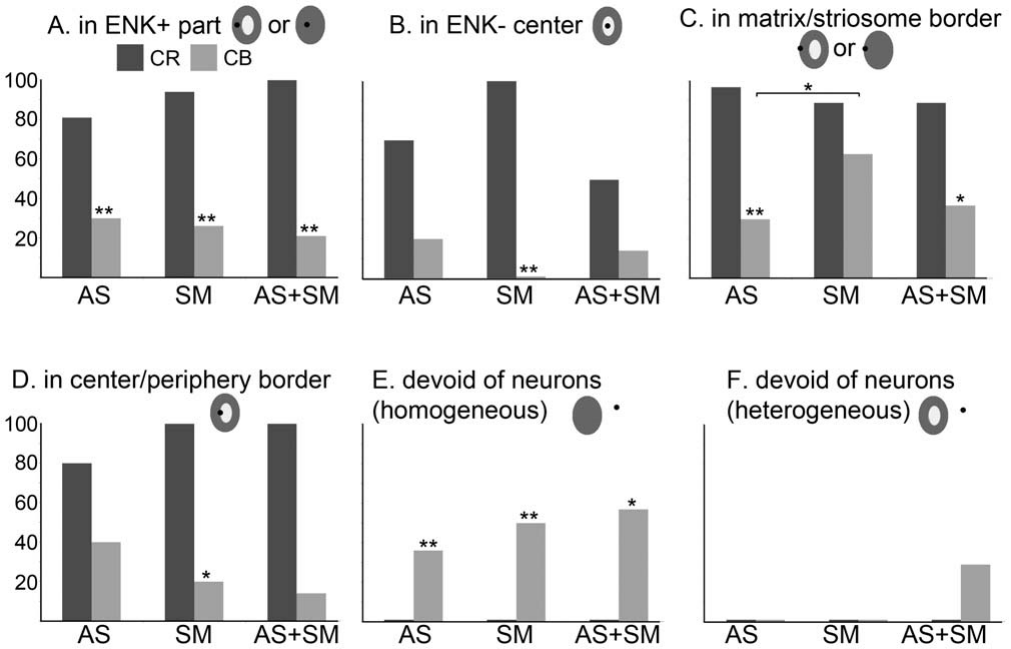
Compartmental organization of the CR- and CB-ir interneurons with respect to the ENK-ir striosomes. Bar graphs showing the percentage of sampled striosomes (Y axis) that contained CR or CB interneurons in the associative (AS), sensorimotor (SM) and associative+sensorimotor overlapped (AS+SM) territories. Each graph refers to the percentage of striosomes that contained interneurons in the mentioned locations, namely: in the dark ENK-ir striosomal aspect (A), in the light ENK-ir striosomal center (B), in the matrix/striosome border (C), in the center/periphery border (D), devoid of cells (striosomes with a homogeneous ENK+ labeling, E), devoid of cells (striosomes with visible center and periphery, F). A total of 58 (for CR; 42 homogeneous, 16 heterogeneous) and 70 (for CB; 53 homogeneous, 17 heterogeneous) striosomes were selected among all the human brains included in the study. Please note that locations A and C include both homogeneous and heterogeneous striosomes. Asterisks show significant (*, 0.05>P>0.01) or highly significant (**, P<0.01) differences between CR and CB interneurons after Fisher's exact test. The only statistical difference between territories within a neuronal group is shown in C (associative vs sensorimotor for CB interneurons).

ENK immunohistochemistry revealed striosomes in every striatal region but the tail of CN and the posteroventral Put, as it has been previously described [33]. Some of the striosomes were composed of a dark ENK-stained periphery and a lightly labeled center (heterogeneous striosomes), although striosomes in the body and gyrus of CN were all homogeneously dark ENK-labeled (homogeneous striosomes). Figure 5 summarizes the main descriptive results of the compartmental distribution of these two interneuronal populations. We have not included the PV- and TH-ir cells in this part of the study because of their low density.

Within each neuronal group, we only found a significant difference in the percentage of striosomes in contact with interneurons between the functional territories of the striatum. This difference, revealed by the Fisher's exact test, was found between the associative and sensorimotor territories when the proportion of striosomes with CB-ir interneurons in the matrix/striosome border was analyzed (associative: 9 striosomes with interneurons in this location vs 21 devoid of them; sensorimotor: 12 vs 7; P = 0.03; Figure 5C). These results confirm the importance of CB-ir interneurons in the integration of the sensorimotor and limbic information.

Comparing between CR- and CB-ir groups, most of the sampled striosomes contained CR-ir interneurons, irrespective to their location inside the striosomal compartment; however, these percentages were below 50% for CB interneurons, except for those patches containing neurons in the matrix/striosome border included in the sensorimotor striatum (Figure 5). Fisher's exact tests comparison between groups revealed differences between neuronal populations in most of functional territories and locations (Figure 5).

## Discussion

The present study is intended to clarify the main anatomical and topographical features of the GABAergic interneurons and dopaminergic cells in the human striatum, as a necessary step to understand the role of these neurons in striatal functioning. For that purpose, we have analyzed the size of their cell bodies, and their distribution and compartmental organization with respect to the matrix/striosomes in the three functional striatal territories (associative, sensorimotor and limbic). The main conclusions are as follows: 1) the volume of their soma is rather constant in the striatal subdivisions, except for the small CR-ir and the PV-ir interneurons, which were significantly smaller in the associative domain; 2) taking all groups of cells together, interneurons are much more abundant in the associative than in the sensorimotor or limbic territories, being this effect driven by CR-ir cells; 3) PV-ir cells are the only group significantly more abundant in the sensorimotor than in the associative striatum; 4) the scarcity of TH-ir cells casts doubts about their importance in the striatum of healthy individuals; 5) CR-ir interneurons are likely to integrate the limbic information channeled through striosomes with both the associative and sensorimotor projections; however, CB-ir interneurons especially attune striosomal projections with the sensorimotor information of the postcommissural Put.

Interneurons play a critical role in the functional organization of the human striatum [23], which in turn is extremely important in many different cognitive, motor and limbic processes. The participation of the striatum in reward-related and goal-directed behavior (see, for example, [47], [48]) demonstrates that this component of the basal ganglia not only has to process effectively the three neural ‘channels’ that is receiving from the cerebral cortex and other subcortical structures, but it also needs to integrate them to generate a proper answer. This integration is extrinsically achieved by means of connections between the different nuclei of the basal ganglia and the thalamus [7]. In addition, the intrinsic regulation by striatal interneurons is equally important [22], [23], [35], [49], [50].

We would like to highlight some methodological remarks about the labeling and classification of the neuronal groups included in the study. The CR immunohistochemical technique reveals different types of interneurons. In earlier reports, they have been classified as medium and large according to the size of their soma [51]. In the present report, we have allocated them into three groups due to the heterogeneity of the previously considered ‘medium’ CR-ir cells. The different mean volumes of the medium and small groups reported here, together with the low dispersion pointed by the SEM, indicate the adequacy of the division into three groups. It should be noted that CR-ir large interneurons have been reported to overlap, at least in part, with striatal cholinergic interneurons [12]. Although we have not performed double-immunostaining to confirm the colocalization of CR and cholinergic markers, the present study and our previous results [17] point to this partial overlapping, since both groups have similar volumes, distribution and compartmental organization. PV-ir neurons represent a quite homogeneous group in terms of their morphology. However, the immunolabeling for CB displays two groups of neurons with different shape and functionality [24], [52], [53]. One of these groups consists of a subset of lightly labeled spiny neurons that profusely occupy the CN and Put, whereas the other one is composed of few medium-sized darkly stained aspiny neurons, scattered mainly in the postcomissural Put. The former are considered projection neurons and the latter interneurons [12], [24]. As it has been mentioned above (*Results* section, *Volume of the cell body* subsection), we propose a reliable method to identify a CB-ir cell as projection neuron or interneuron according to its anatomical features. One other method to identify CB-ir cells as projection neurons or interneurons would have been double immunostaining with ENK or dynorphin, both of them specific markers of projection neurons. In the present study, due to the high amount of processed slices required for the stereological analysis, we opted for a morphological criterion. About the size of this interneuronal population, their soma has been reported to be larger in previous descriptions [12]. This discrepancy could be due to a different processing (i.e. fixation) of the brain material before the immunohistochemistry, which is known to cause some shrinkage. The GABAergic nature of the CB-ir interneurons has not been confirmed yet, although there are several evidences that may lead to think that they release this inhibitory neurotransmitter. Firstly, the CB-ir projection neurons are undoubtedly GABAergic [53]; secondly, the other groups of striatal interneurons that express calcium binding proteins (i.e. CR- and PV-ir) release GABA [23], [50]; and, finally, about 40% of the CB-ir interneurons colocalize with NADPH-positive intrinsic neurons, that are considered nitrergic and GABAergic [22], [54].

TH immunohistochemistry is considered the most reliable method to identify dopaminergic cells in the human striatum [16], [55], [56], both in normal control individuals and Parkinson's disease patients. Their role as interneurons has been suggested according to their morphological features, and recently they have been shown to be well-integrated elements of striatal circuitry [57], [58]. Several studies to disentangle the co-expression of different markers and neurotransmitters in this neuronal population have been reported. In humans, most of them express GABA markers, as well as other dopaminergic markers (dopamine transporter, DAT) [16]. In rodents, TH neurons also express dynorphin and ENK, suggesting that they are projection neurons of the direct and indirect striatal output pathways [59]. As it has been mentioned above for the CB-ir interneurons, we have not performed double-labeling of this neuronal population due to methodological issues.

The stereological approach used in the present study provides an accurate estimation of neuronal density, that is, number of cells per volume unit. In our opinion, this quantitative variable is more informative than the total number of cells, since our main goal is to compare regions with very different sizes. As it has been discussed before [17], postmortem brain shrinkage could inflate the actual numbers obtained with these methods, but the comparison between the different striatal regions, which is the core of the study, would not be affected. The number of subjects included in the study does not allow us to weight the possible effects of gender or age in neuronal density. In any case, the range of age (from 20 to 76 years, see Table 1) suggests that this effect could be somewhat compensated.

Our previous research on nitrergic and cholinergic interneurons [17], [18] indicated a negative correlation between the size of the cell body and the density (cells/mm^3^) in the striatal subdivisions. A similar trend has also been found in the present study for the most abundant subset of interneurons, which are the small CR-ir ones. Despite the fact that this correlation was positive (i.e. larger neurons where they are more numerous) for the PV-ir interneurons, we hypothesize that overall interneurons may tend to have a larger cell body volume where they are fewer. The singular case observed in PV-ir interneurons might respond to that: 1) their density is low (101 cells/mm^3^) even in the striatal territory where they are more numerous (sensorimotor); 2) the effect on size may depend on the global neuronal density in the area, and not only the abundance of a particular cell type; in that case, the sensorimotor territory contains much fewer cells than the associative one.

To our knowledge, this is the first research work to report systematically the density of the GABAergic interneurons and dopaminergic cells in the non-pathological human striatum. Together with our previous studies, it proves the abundance of local circuit neurons in those regions involved in high cognitive processes. Their recruitment in motor or limbic tasks does not seem to be so critical, attending to the difference in density. Although we do not present here the neuronal density in anatomical territories (divided in the anteroposterior, dorsoventral and mediolateral axes), it is worth noting that these groups of cells are in general most abundant in the posterior aspects of CN and Put, similarly to nitrergic and cholinergic cells. Because of their small size, these regions are frequently neglected in animal connectivity research, as well as in Neuroimaging human studies. The present report demonstrates the potential importance of the most posterior part of the striatum, especially of the CN, in cognitive tasks. It should be taken into account, however, that striatal interneurons have a widespread axonal arborization and, although few, can influence a high number of striatal neurons. We here assume that the axonal arborization of a particular interneuronal group is constant across the striatum, although this hypothesis should be confirmed in the future.

In our opinion, the analysis of the neuronal distribution we have carried out gives two functional insights. On the one hand, by investigating the distribution in the territories with a ‘single’ pattern of connections, we are able to know whether a group of neurons is involved in processing that type of neural information (‘within territories’ integration). On the other hand, by analyzing those regions with overlapped projections, we can make assumptions about the implication of each group integrating both types of information (‘between territories’ integration). Therefore, our results emphasize the importance of CR-ir GABAergic interneurons handling associative information, whereas PV-ir GABAergic cells are recruited for sensorimotor tasks. Furthermore, CR-ir neurons are equally involved in the integration of the associative neural information with either the sensorimotor or limbic one, although PV- and CB-ir interneurons are more specialized in the integration of associative and sensorimotor projections.

Another striking finding of the present study is the relative scarcity of CB-ir cells in the sensorimotor striatum, since they were thought to be abundant in the postcommissural Put [12]. This controversy could be due to the differential immunolabeling of these interneurons in the CN and Put, especially in its lateral half. Interestingly, and in all brains and sections included in this study, CB-ir interneurons were more darkly stained in the lateral aspects of the Put than in the CN. This can be a confounding factor to believe that this subset of interneurons is more abundant in the postcommissural Put than in any other striatal region. Thanks to the anatomical criteria we mentioned above, we could develop a reliable method to identify CB immunolabeled interneurons. Whether the darkest labeling in the lateral Put points to a higher amount of CB inside the cell is still to be determined, and it could be of importance to evaluate the reaction of these interneurons against abnormal increased levels of intracellular calcium.

Since the first descriptions of the striosomal compartment [25], there has been a growing interest in the anatomy and connectivity of this three-dimensional striatal structure. Its neurochemical characteristics have been thoroughly described in rodents [60], [61] and humans [26], [27], [33]. The hodological data collected in animal research with tract-tracing methods indicate that it is a limbic-related compartment located in an associative or sensorimotor matrix, and whose projection neurons target structures such as the substantia nigra pars compacta that are not innervated by the matrix [28], [30], [31], [32], [62]. Therefore, those interneurons located in the matrix/striosome border could integrate different types of input and output neural information, as it has been suggested by other groups [49], [63]. The abundance of CR-ir interneurons at the border of the two main striatal compartments suggests a large-scale integrative role of these cells. However, CB-ir intrinsic neurons are scarce at the matrix/striosome boundaries in the associative striatum, which may indicate a selective task in the integration of sensorimotor (matrix) and limbic (striosomes) connections. Interestingly, this has been also pointed out for PV-ir interneurons in rats with a completely different methodology [64]. In the present study, we decided to rule out the analysis of the compartmental distribution of PV- and TH-ir cells. We found that striosomes seldom contained these interneurons, which in addition were scarce when present (usually one single cell). For that reason, we here argue their role in the integration of the matrix/striosome information.

Finally, the present report can shed some light regarding the origin and development of neuropsychiatric pathological processes that affect the striatum, such as Huntington's disease. The massive loss of striatal projection neurons [12], [53], [65] that, as mentioned above, are CB-ir, is due to an abnormal increase of calcium within the cell [66]. Conversely, interneurons are relatively spared in this pathology [12], [67], [68]. As a possible explanation of this fact one might think in the neuroprotective role of calcium-binding proteins, which appear to be greatly accumulated in CB-ir interneurons located in the lateral postcommissural Put. It would be interesting to assess whether lightly stained CB-ir interneurons, preferentially located in the associative striatum, are also preserved or they follow the same fate as the projection neurons. If the latter is true, it could account in part for the cognitive impairment caused by this disease. Concerning another group of GABAergic interneurons, a recent research shows that a decrease of PV-ir interneurons in the striatum of individuals with Tourette syndrome could be associated with the motor impairment caused by such disease [69]. This finding goes in agreement with the present study, which suggests that this chemospecific group of interneurons may have an important role in the integration of motor information. In a completely different scenario, TH-ir cells seem to be more abundant in pathological states, such as Parkinson's disease, than in postmortem material collected from control subjects. It could be argued that the apparent lack of these cells in our results might be due to an unsuccessful immunostaining of the sections. However, we did find many TH-ir cells in the substantia nigra *pars compacta* and VTA in the same slices (data not shown), and the low numbers we found in the striatum are in agreement with previous research [55], [56]. It has been proposed that these neurons may compensate the lack of mesostriatal dopamine that occurs in Parkinson's disease [16], [56], [70]. However, a more recent study demonstrated a decrease of these cells in both Parkinson's and Huntington's disease, compared with controls [55]. The authors discussed that L-Dopa treatment could be the cause of this decrease, and actually demonstrated it in a further study in parkinsonian monkeys treated with this drug [71]. Whatever might be their role in pathological states, the low density of dopaminergic cells reported here casts doubts about their influence in the integration of neural information.

In conclusion, our results show the preferential role of PV-ir and CB-ir interneurons in the processing of the striatal sensorimotor information and its integration with the limbic projections of the striosomes. In addition, we demonstrate that interneurons are much more abundant in the associative territory, due to the large amount of CR-ir cells that are likely to integrate associative with either somatosensory or limbic information. The dopaminergic neurons are scarce in every striatal territory. This report may help understand the functional organization of the normal human striatum, and provide some new ideas in the study of pathological states.

## References

[1] Monchi O, Petrides M, Petre V, Worsley K, Dagher A (2001). Wisconsin Card Sorting revisited: distinct neural circuits participating in different stages of the task identified by event-related functional magnetic resonance imaging.. J Neurosci.

[2] Lehericy S, Bardinet E, Tremblay L, Van de Moortele PF, Pochon JB (2006). Motor control in basal ganglia circuits using fMRI and brain atlas approaches.. Cereb Cortex.

[3] Haber SN, Kim KS, Mailly P, Calzavara R (2006). Reward-related cortical inputs define a large striatal region in primates that interface with associative cortical connections, providing a substrate for incentive-based learning.. J Neurosci.

[4] Parent A (1990). Extrinsic connections of the basal ganglia.. Trends Neurosci.

[5] Fudge JL, Kunishio K, Walsh P, Richard C, Haber SN (2002). Amygdaloid projections to ventromedial striatal subterritories in the primate.. Neuroscience.

[6] Alexander GE, DeLong MR, Strick PL (1986). Parallel organization of functionally segregated circuits linking basal ganglia and cortex.. Annu Rev Neurosci.

[7] Haber SN (2003). The primate basal ganglia: parallel and integrative networks.. J Chem Neuroanat.

[8] Lopez de Maturana R, Sanchez-Pernaute R (2010). Regulation of Corticostriatal Synaptic Plasticity by G Protein-Coupled Receptors.. CNS Neurol Disord Drug Targets.

[9] Flores-Barrera E, Vizcarra-Chacon BJ, Tapia D, Bargas J, Galarraga E (2010). Different corticostriatal integration in spiny projection neurons from direct and indirect pathways.. Front Syst Neurosci.

[10] Qi Z, Miller GW, Voit EO (2010). The internal state of medium spiny neurons varies in response to different input signals.. BMC Syst Biol.

[11] Kehagia AA, Murray GK, Robbins TW (2010). Learning and cognitive flexibility: frontostriatal function and monoaminergic modulation.. Curr Opin Neurobiol.

[12] Cicchetti F, Prensa L, Wu Y, Parent A (2000). Chemical anatomy of striatal interneurons in normal individuals and in patients with Huntington's disease.. Brain Res Brain Res Rev.

[13] Waldvogel HJ, Billinton A, White JH, Emson PC, Faull RL (2004). Comparative cellular distribution of GABAA and GABAB receptors in the human basal ganglia: immunohistochemical colocalization of the alpha 1 subunit of the GABAA receptor, and the GABABR1 and GABABR2 receptor subunits.. J Comp Neurol.

[14] Wu Y, Parent A (2000). Striatal interneurons expressing calretinin, parvalbumin or NADPH-diaphorase: a comparative study in the rat, monkey and human.. Brain Res.

[15] Bedard A, Gravel C, Parent A (2006). Chemical characterization of newly generated neurons in the striatum of adult primates.. Exp Brain Res.

[16] Cossette M, Levesque D, Parent A (2005). Neurochemical characterization of dopaminergic neurons in human striatum.. Parkinsonism Relat Disord.

[17] Bernacer J, Prensa L, Gimenez-Amaya JM (2007). Cholinergic interneurons are differentially distributed in the human striatum.. PLoS One.

[18] Bernacer J, Prensa L, Gimenez-Amaya JM (2005). Morphological features, distribution and compartmental organization of the nicotinamide adenine dinucleotide phosphate reduced-diaphorase interneurons in the human striatum.. J Comp Neurol.

[19] Graveland GA, Williams RS, DiFiglia M (1985). A Golgi study of the human neostriatum: neurons and afferent fibers.. J Comp Neurol.

[20] Roberts RC, Gaither LA, Peretti FJ, Lapidus B, Chute DJ (1996). Synaptic organization of the human striatum: a postmortem ultrastructural study.. J Comp Neurol.

[21] Levesque M, Parent A (2005). The striatofugal fiber system in primates: a reevaluation of its organization based on single-axon tracing studies.. Proc Natl Acad Sci U S A.

[22] Kawaguchi Y, Wilson CJ, Augood SJ, Emson PC (1995). Striatal interneurones: chemical, physiological and morphological characterization.. Trends Neurosci.

[23] Tepper JM, Bolam JP (2004). Functional diversity and specificity of neostriatal interneurons.. Curr Opin Neurobiol.

[24] Prensa L, Gimenez-Amaya JM, Parent A (1998). Morphological features of neurons containing calcium-binding proteins in the human striatum.. J Comp Neurol.

[25] Graybiel AM, Ragsdale CW (1978). Histochemically distinct compartments in the striatum of human, monkeys, and cat demonstrated by acetylthiocholinesterase staining.. Proc Natl Acad Sci U S A.

[26] Holt DJ, Graybiel AM, Saper CB (1997). Neurochemical architecture of the human striatum.. J Comp Neurol.

[27] Prensa L, Gimenez-Amaya JM, Parent A (1999). Chemical heterogeneity of the striosomal compartment in the human striatum.. J Comp Neurol.

[28] Saka E, Graybiel AM (2003). Pathophysiology of Tourette's syndrome: striatal pathways revisited.. Brain Dev.

[29] Avendano C, de Las Heras S, Gimenez-Amaya JM (2005). Striatal projections from the lateral and posterior thalamic complexes.. An anterograde tracer study in the cat. Histochem Cell Biol.

[30] Eblen F, Graybiel AM (1995). Highly restricted origin of prefrontal cortical inputs to striosomes in the macaque monkey.. J Neurosci.

[31] Prensa L, Parent A (2001). The nigrostriatal pathway in the rat: A single-axon study of the relationship between dorsal and ventral tier nigral neurons and the striosome/matrix striatal compartments.. J Neurosci.

[32] Fujiyama F, Sohn J, Nakano T, Furuta T, Nakamura KC (2011). Exclusive and common targets of neostriatofugal projections of rat striosome neurons: a single neuron-tracing study using a viral vector.. Eur J Neurosci.

[33] Bernacer J, Prensa L, Gimenez-Amaya JM (2008). Chemical architecture of the posterior striatum in the human brain.. J Neural Transm.

[34] Waldvogel HJ, Kubota Y, Fritschy J, Mohler H, Faull RL (1999). Regional and cellular localisation of GABA(A) receptor subunits in the human basal ganglia: An autoradiographic and immunohistochemical study.. J Comp Neurol.

[35] Apicella P (2002). Tonically active neurons in the primate striatum and their role in the processing of information about motivationally relevant events.. Eur J Neurosci.

[36] Centonze D, Gubellini P, Bernardi G, Calabresi P (1999). Permissive role of interneurons in corticostriatal synaptic plasticity.. Brain Res Brain Res Rev.

[37] Alelu-Paz R, Iturrieta-Zuazo I, Byne W, Haroutunian V, Garcia-Villanueva M (2008). A new antigen retrieval technique for human brain tissue.. PLoS One.

[38] Hsu SM, Raine L, Fanger H (1981). Use of avidin-biotin-peroxidase complex (ABC) in immunoperoxidase techniques: a comparison between ABC and unlabeled antibody (PAP) procedures.. J Histochem Cytochem.

[39] Selden N, Geula C, Hersh L, Mesulam MM (1994). Human striatum: chemoarchitecture of the caudate nucleus, putamen and ventral striatum in health and Alzheimer's disease.. Neuroscience.

[40] Schaltenbrand G, Wahren W (1977). Atlas for stereotaxy of the human brain..

[41] Mai J, Assheuer J, Paxinos G (1997). Atlas of the human brain..

[42] Nowinski W, Bryan R, Raghavan R (1997). The electronic clinical brain atlas..

[43] Mayhew TM, Gundersen HJ (1996). If you assume, you can make an ass out of u and me': a decade of the disector for stereological counting of particles in 3D space.. J Anat.

[44] Bjugn R, Gundersen HJ (1993). Estimate of the total number of neurons and glial and endothelial cells in the rat spinal cord by means of the optical disector.. J Comp Neurol.

[45] Wreford NG (1995). Theory and practice of stereological techniques applied to the estimation of cell number and nuclear volume in the testis.. Microsc Res Tech.

[46] Martin R, Fraile B, Peinado F, Arenas MI, Elices M (2000). Immunohistochemical localization of protein gene product 9.5, ubiquitin, and neuropeptide Y immunoreactivities in epithelial and neuroendocrine cells from normal and hyperplastic human prostate.. J Histochem Cytochem.

[47] Haber SN, Knutson B (2010). The reward circuit: linking primate anatomy and human imaging.. Neuropsychopharmacology.

[48] Grahn JA, Parkinson JA, Owen AM (2009). The role of the basal ganglia in learning and memory: neuropsychological studies.. Behav Brain Res.

[49] Saka E, Iadarola M, Fitzgerald DJ, Graybiel AM (2002). Local circuit neurons in the striatum regulate neural and behavioral responses to dopaminergic stimulation.. Proc Natl Acad Sci U S A.

[50] Tepper JM, Koos T, Wilson CJ (2004). GABAergic microcircuits in the neostriatum.. Trends Neurosci.

[51] Cicchetti F, Beach TG, Parent A (1998). Chemical phenotype of calretinin interneurons in the human striatum.. Synapse.

[52] Bennett BD, Bolam JP (1993). Two populations of calbindin D28k-immunoreactive neurones in the striatum of the rat.. Brain Res.

[53] Kiyama H, Seto-Ohshima A, Emson PC (1990). Calbindin D28K as a marker for the degeneration of the striatonigral pathway in Huntington's disease.. Brain Res.

[54] Kawaguchi Y (1997). Neostriatal cell subtypes and their functional roles.. Neurosci Res.

[55] Huot P, Parent A (2007). Dopaminergic neurons intrinsic to the striatum.. J Neurochem.

[56] Porritt MJ, Batchelor PE, Hughes AJ, Kalnins R, Donnan GA (2000). New dopaminergic neurons in Parkinson's disease striatum.. Lancet.

[57] Ibanez-Sandoval O, Tecuapetla F, Unal B, Shah F, Koos T (2010). Electrophysiological and morphological characteristics and synaptic connectivity of tyrosine hydroxylase-expressing neurons in adult mouse striatum.. J Neurosci.

[58] Unal B, Ibanez-Sandoval O, Shah F, Abercrombie ED, Tepper JM (2011). Distribution of tyrosine hydroxylase-expressing interneurons with respect to anatomical organization of the neostriatum.. Front Syst Neurosci.

[59] Darmopil S, Muñetón-Gómez VC, de Ceballos VL, Bernson M, Moratalla R (2008). Tyrosine hydroxylase cells appearing in the mouse striatum after dopamine denervation are likely to be projection neurones regulated by L-DOPA.. Eur J Neurosci.

[60] Graybiel AM (1984). Correspondence between the dopamine islands and striosomes of the mammalian striatum.. Neuroscience.

[61] Graybiel AM, Chesselet MF (1984). Compartmental distribution of striatal cell bodies expressing [Met]enkephalin-like immunoreactivity.. Proc Natl Acad Sci U S A.

[62] Prensa L, Gimenez-Amaya JM, Parent A, Bernacer J, Cebrian C (2009). The nigrostriatal pathway: axonal collateralization and compartmental specificity.. J Neural Transm Suppl.

[63] Martone ME, Young SJ, Armstrong DM, Groves PM (1994). The distribution of cholinergic perikarya with respect to enkephalin-rich patches in the caudate nucleus of the adult cat.. J Chem Neuroanat.

[64] Ramanathan S, Hanley JJ, Deniau JM, Bolam JP (2002). Synaptic convergence of motor and somatosensory cortical afferents onto GABAergic interneurons in the rat striatum.. J Neurosci.

[65] Ferrante RJ, Kowall NW, Richardson EP (1991). Proliferative and degenerative changes in striatal spiny neurons in Huntington's disease: a combined study using the section-Golgi method and calbindin D28k immunocytochemistry.. J Neurosci.

[66] Pandey M, Mohanakumar KP, Usha R (2010). Mitochondrial functional alterations in relation to pathophysiology of Huntington's disease.. J Bioenerg Biomembr.

[67] Dawbarn D, De Quidt ME, Emson PC (1985). Survival of basal ganglia neuropeptide Y-somatostatin neurones in Huntington's disease.. Brain Res.

[68] Ferrante RJ, Kowall NW, Beal MF, Martin JB, Bird ED (1987). Morphologic and histochemical characteristics of a spared subset of striatal neurons in Huntington's disease.. J Neuropathol Exp Neurol.

[69] Kataoka Y, Kalanithi PS, Grantz H, Schwartz ML, Saper C (2010). Decreased number of parvalbumin and cholinergic interneurons in the striatum of individuals with Tourette syndrome.. J Comp Neurol.

[70] Betarbet R, Turner R, Chockkan V, DeLong MR, Allers KA (1997). Dopaminergic neurons intrinsic to the primate striatum.. J Neurosci.

[71] Huot P, Levesque M, Morissette M, Calon F, Dridi M (2008). L-Dopa treatment abolishes the numerical increase in striatal dopaminergic neurons in parkinsonian monkeys.. J Chem Neuroanat.

